# Evaluation of oral health-related quality of life and its association with mental health status of patients with type 2 diabetes mellitus in the post-COVID-19 pandemic era: A study from Central Saudi Arabia

**DOI:** 10.3389/fpubh.2023.1158979

**Published:** 2023-03-24

**Authors:** Ashokkumar Thirunavukkarasu, Majed Sonitan Alharbi, Mohammad Salahuddin, Ahmad Homoud Al-Hazmi, Bashayer Farhan ALruwaili, Aseel Awad Alsaidan, Ahmad Saeed Almutairi, Rayyanah Nasser Almuhaydib, Latifah Ibrahim Alrashoudi

**Affiliations:** ^1^Department of Community and Family Medicine, College of Medicine, Jouf University, Sakaka, Aljouf, Saudi Arabia; ^2^Health Care Delivery Department, Qassim Health Cluster, Buraidah, Qassim, Saudi Arabia; ^3^Department of Physiology, College of Medicine, Jouf University, Sakaka, Aljouf, Saudi Arabia; ^4^Commitment Department, Ministry of Health, Directorate of Health Affairs, Riyadh, Saudi Arabia

**Keywords:** depression, diabetes, oral health, quality of life, Saudi Arabia

## Abstract

**Background and objectives:**

The association between oral and mental health is reciprocal, in which poor oral health may lead to several mental health issues, especially among patients with diabetes. The present study evaluated oral health-related quality of life (OHRQOL) and its association with mental health conditions among patients with type 2 diabetes mellitus (T2DM) in central Saudi Arabia.

**Methods:**

The Arabic version of the Oral Health Impact Profile-14 (OHIP-14) questionnaire and the Depression, Anxiety, and Stress Scale-21 Items (DASS-21) were used to assess the OHRQOL and mental health status of patients with diabetes. We utilized logistic regression analysis to identify the predictors of poor OHRQOL, and Spearman’s correlation test to identify any correlations between OHIP-14 and overall DASS-21 scores, as well as each subscale.

**Results:**

Of the 677 patients included in the present study, 52.7% had a poor OHRQOL, which was significantly higher (positive association) among patients with a longer duration of diabetes (adjusted odds ratio [AOR] = 3.31; 95% confidence interval [CI] = 1.96–4.17) and those who did not periodically monitor their oral health (AOR = 2.85; 95% CI = 1.76–3.89). Some forms (mild, moderate, severe, or extremely severe) of depression, anxiety, and stress were observed in 59.7, 71.1, and 67.1% of the participants, respectively. Furthermore, we found that the total OHRQOL scores had a significant positive association with depression (AOR = 2.32, 95% CI = 1.34–3.71, *p* = 0.001), anxiety (AOR = 1.81, 95% CI = 1.22–2.79, *p* = 0.003), and stress (AOR = 1.43, 95% CI = 1.14–2.19, *p* = 0.026).

**Conclusion:**

The results of the present study suggest the importance of appropriate and targeted health education programs for T2DM patients to ensure periodic dental examinations and oral health. Additionally, we recommend counseling sessions for all T2DM patients with trained healthcare providers to improve their mental health status during follow-up visits at outpatient diabetes care centers.

## 1. Introduction

The incidence of diabetes mellitus (DM) is rapidly increasing worldwide, especially in low- and middle-income countries. As such, DM is emerging as a major public health issue worldwide, including in the Kingdom of Saudi Arabia (KSA) (1, 2). The COVID-19 pandemic has amplified this global burden, as several studies have reported an increased risk of developing diabetes among individuals who had suffered from severe acute respiratory syndrome coronavirus - 2 (SARS-CoV-2) infection (3, 4).

According to the World Health Organization (WHO), good oral health is an essential marker for general health, mental health, and health-related quality of life (5, 6), and includes tooth decay (dental caries), gum (periodontal) diseases, oral injuries, and oral cancer (6, 7). Oral health and oral health-related quality of life (OHRQOL) share several modifiable risk factors for chronic non-communicable diseases (i.e., DM, cardiovascular diseases, and cancer) (5, 8). Additionally, causal associations have been documented between DM and poor oral health (9, 10). As reported in previous studies, poor oral health eventually leads to a poor OHRQOL (10, 11), although the association between oral and mental health is reciprocal. As such, poor oral health may in turn lead to several mental health issues, such as depression, anxiety, phobia of dental treatment, or affective and/or eating disorders. Additionally, there is a prevalence of dental caries, periodontitis, tooth loss, and improper dental-care utilization in those with poor oral health (12, 13). Similarly, previous evidence has suggested that poor oral health and psychiatric disorders are associated with the development of DM, and that patients with DM are two-to-three times more at risk of developing depression than those without DM. Fewer than half of the individuals with DM who have depression and other mental disorders, however, are diagnosed and receive adequate treatment (14, 15).

During the COVID-19 pandemic, people with diabetes reported several oral health problems due to limited access to dental care, fear factors and a shortage of dental equipment and supplies (16–18). One year after the first lockdowns, dentists worldwide reported the adverse effects of the COVID-19 pandemic on oral health, such as a higher incidence of advanced periodontal diseases and tooth decay that led to decreased OHRQOL (19, 20). A study published by Ciardo et al. during the COVID -19 era reported that OHRQOL was significantly associated with the mental health status of their participants (21). Several authors stated that concerns persist regarding the long-term health effects of the COVID-19 pandemic, and its potentially significant mental health consequences are still poorly understood, especially among people with chronic illness (22, 23). A survey by Khalifa et al., which assessed OHRQOL among patients with and without Type 2 DM(T2DM) in the United Arab Emirates, indicated that among patients with T2DM, OHRQOL was significantly associated with both the social disability and handicap domains. In contrast, the handicap domain was the only domain significantly associated with non-diabetic patients (24). Another study, performed during the COVID-19 pandemic by Moradian et al., found a significant increase in the prevalence of psychiatric symptoms, namely depression, generalized anxiety, and distress, among patients with T2DM after the COVID-19 outbreak. Increased depression-related symptoms, generalized anxiety, and distress were predicted by COVID-19-related fear, whereas only higher depressive symptoms were predicted by trust in governmental actions to face COVID-19 (25).

The COVID-19 pandemic has significantly impacted the short- and long-term mental health of numerous individuals, and T2DM patients are no exception (22, 26, 27). Considering the high prevalence of T2DM in the KSA and poor oral health among them, it is critical to evaluate OHRQOL, mental health status, and predisposing factors among T2DM patients, especially in the post-COVID-19 pandemic era (28, 29). Appropriate healthcare services, therefore, can be implemented at diabetes care centers for prompt diagnosis and necessary referrals. Furthermore, necessary interventions targeting mental health among T2DM patients can be designed to improve OHRQOL and mental health simultaneously. The available literature on this subject in KSA, however, is limited. Therefore, the present study was conducted to evaluate the OHRQOL, mental health status, and associated factors among patients with T2DM in the Qassim region of KSA. We also assessed the predictive effect of T2DM patients’ OHRQoL on their mental health status.

## 2. Participants and methods

### 2.1. Research design

The present quantitative cross-sectional study was conducted from August 2022 to January 2023.

### 2.2. Research setting

The present study was conducted in the Qassim (central) region of the KSA, one of the 13 provinces in the country, with a total population of approximately 1.4 million. In the KSA, outpatient diabetes care is provided at primary health centers (PHCs) and diabetic clinics associated with the Ministry of Health.

### 2.3. Inclusion and exclusion criteria

The inclusion criteria were as follows: patients with T2DM, 18–65 years of age, attending outpatient diabetes care facilities of the Ministry of Health in the Qassim region of the KSA, and with duration of diabetes ≥1 year. The exclusion criteria were as follows: other types of DM patients pediatric patients, hospital inpatients with T2DM, and those unwilling to participate.

### 2.4. Sampling strategy

The required number of patients with T2DM for analysis in the present study was estimated using Cochran’s equation (z^2^pq/e^2^), where p was the expected prevalence of 73% for some degree of depression among patients with T2DM, as stated by Aljohani et al. in 2021 (30), q was 1–0.73, and e was the margin of error at 5%. We applied all stated values to the equation and found that the minimum required sample size was 303. Furthermore, considering two different settings (diabetes clinics and PHCs) and a 10% additional sample size, the research team concluded that the required minimum sample size was 677. The present study used a consecutive sampling method to obtain the required number of patients. Using this technique, we consecutively invited every 5th T2DM patient from the outpatient diabetes care facilities to participate in the study until we obtained the minimum required sample (*n* = 677). A total of 805 participants were invited for the present study. Every 5th patient recruitment strategy was selected after a focus group discussion with the physicians at the diabetes care facilities to avoid the possibility of patients from the same family sharing the same sociodemographic background characteristics. Furthermore, we restricted the maximum per day to 10 patients from each type of facility to enroll patients over a longer period of time.

### 2.5. Data collection method

First, we explained the purpose of the present study to patients with T2DM, and obtained informed consent from those who were willing to participate. We collected anonymized participant data using an Arabic version of the survey, and validated the data collection protocol, which consisted of three sections. The first section gathered data regarding the participants’ age, gender, smoking status, educational status, occupational status, associated comorbidities, and oral health-related behaviors. The second section consisted of the administration of the Oral Health Impact Profile-14 (OHIP-14) questionnaire, which is a validated and reliable tool (Cronbach’s alpha (α) > 0.70) used to evaluate OHRQOL in a variety of settings, including Arab countries (31–33). The psychometric properties of the Arabic version of the OHIP-14 are described below. Cronbach’s α, which measures internal consistency, was 0.89 (high). Additionally, the test–retest correlation coefficient was highly acceptable for each item (0.81–0.97) and subscale score (0.85–0.97). These values indicate that the OHIP-14 is reproducible and can be used in a variety of settings. Furthermore, the OHIP-14 has adequate internal consistency and discriminant validity for all subscales. The OHIP-14 assesses 7 domains, including functional constraints, physical pain, psychological discomfort, physical disability, psychological disability, social disability, and handicap, using 14 questions. The study patients used a 5-point Likert scale, ranging from “never” to “very often,” to answer all 14 questions, and we scored each item from 0 to 4, accordingly. We combined all domains and categorized them as having a poor or good OHRQoL, according to the threshold demarcation value formula [(total highest score − total lowest score)/2 + total lowest score]. The highest total OHIP-14 score was 54, and the lowest total score was 6. The cutoff value, therefore, was set as 30 to categorize patients as having a poor (≥30) or good (<30) OHRQOL. Furthermore, a higher total OHIP-14 score indicates poorer OHRQoL. The third and final section consisted of the Depression, Anxiety, and Stress Scale-21 Items (DASS-21), a self-reported scale utilized to evaluate the emotional states of depression, anxiety, and stress. Each of the DASS-21 subscales is comprised of seven items. Similar to the OHIP-14, the DASS-21 scale is a validated and reliable tool used in a variety of settings worldwide, including in Arab countries. The Arabic version of the DASS-21 tool showed high internal consistency (α = 0.94), and the internal reliability coefficients for the depression, anxiety, and stress subscales were 0.88, 0.81, and 0.89, respectively (34–37). The study patients responded on a 4-point Likert scale, ranging from “did not apply to me at all” (score, 0) to “applied to me most of the time” (score, 3). The total DASS-21 scores and each subscale were combined, and categorized as normal, mild, moderate, severe, or extremely severe (36, 38).

### 2.6. Data analysis

The research team used the Statistical Package for Social Science (SPSS, version 21) to code and analyze the anonymized data of patients with T2DM related to oral health and mental health. Descriptive results are presented as frequencies, proportions, means, standard deviations (SDs), medians, and interquartile ranges (IQRs). We utilized binary logistic regression analysis (poor vs. good) to identify predictors of poor OHRQoL and to find the predictive effect of OHRQOL on the mental health of T2DM patients. An adjusted odds ratio (AOR) with a confidence interval (CI) that did not include a null value of one was considered a significant predictor. During the data curation and normality assumption analysis, we found that the OHIP-14 and DASS-21 scores did not meet the normality assumption (Shapiro–Wilk test). We, therefore, utilized the Kruskal-Wallis (for categorical variables), Mann–Whitney U (for dichotomous variables), and Spearman’s correlation tests to identify the correlations between the OHIP-14 scores and the total DASS-21 scores as well as each subscale. The significance (*p* < 0.05) value was interpreted based on two-tailed tests.

## 3. Results

During the data collection period, 805 eligible patients with T2DM were contacted, of whom 677 (required sample size for the present study) consented to participate in the present survey (response rate 84.09%).

Of the 677 patients, the majority (44.2%) were 40–50 years old, males (52.9%), studied at the university level (62.0%), married (89.2%), had a monthly income >7,000 Saudi Riyal (SAR [1 USD = 3.75 SAR]) (42.1%), and were non-smokers (78.4%). When assessing oral health-related behaviors, 57.9% brushed their teeth at least once per day, although approximately one-fourth (23.8%) missed periodic dental check-ups and 18.3% perceived their oral health as either poor or fair (Table 1).

**Table 1:** Background and oral health related characteristics of the study population (*n* = 677).

Characteristics	Number	%
Age (mean ± SD)	46.7 ± 9.2	
Less than 40 years	169	25.0
40 to 50 years	299	44.2
More than 50 years	209	30.9
**Gender**		
Male	358	52.9
Female	319	47.1
**Occupation** ^**#**^		
Government	171	25.3
Private	388	57.3
Unemployed	118	17.4
**Education level**		
Up to high school	257	38.0
University (UG and PG)	420	62.0
**Marital status**		
Married	604	89.2
Single	73	10.8
**Monthly income (1 USD = 3.75 SAR)**		
Up to 5,000 SAR	109	16.1
5,000 to 7,000 SAR	283	41.8
More than 7,000 SAR	285	42.1
**Presence of other chronic illness**		
No	454	67.1
Yes	223	32.9
Duration of Diabetes (mean ± SD)	8.32 ± 4.5	
**Smoking status (including shisha)**		
No	531	78.4
Yes	146	21.6
**Brushing teeth (per day)**		
Once or less	285	42.1
Twice or more	392	57.9
**Dental health checkup by the dentist (every 6 months)**		
No	161	23.8
Yes	516	76.2
**Perception on their oral health**		
Excellent	45	6.6
Very good	197	29.1
Good	311	45.9
Fair	121	17.9
Poor	3	0.4

# Government – Currently working in any of the government sector departments of KSA; private - currently working in private sectors, self-employed/business; Unemployed – Currently not working in any job.

When looking at the data analyzed related to the OHIP-14 profile, more than one-third (36.7%) of the participants often (fairly and very) had worsened taste, and 11.4% very often had discomfort in eating daily food. Similarly, 1.3% of participants very often felt embarrassed of their oral health (Table 2).

**Table 2:** Diabetes patients responses related to oral health impact profile – 14 (OHIP – 14) assessment (*n* = 677).

Domains	Items	Never *n* (%)	Hardly ever *n* (%)	Occasionally *n* (%)	Fairly often *n* (%)	Very often *n* (%)
Functional limitations	Trouble pronouncing words	22 (3.2)	40 (5.9)	139 (20.5)	283 (41.8)	193 (28.5)
	Worsened sense of taste	4 (0.6)	134 (19.8)	290 (42.8)	174 (25.7)	75 (11.1)
Physical pain	Painful aching in mouth	4 (0.6)	137 (20.2)	282 (41.7)	163 (24.1)	91 (13.4)
	Uncomfortable to eat food	17 (2.5)	114 (16.8)	265 (39.1)	204 (30.1)	77 (11.4)
Psychological discomfort	Being self-conscious	26 (3.8)	131 (19.4)	223 (32.9)	213 (31.5)	84 (12.4)
	Feeling tense	22 (3.2)	128 (18.9)	264 (39.0)	200 (29.5)	63 (9.3)
Physical disability	Unsatisfactory diet	23 (3.4)	142 (21.0)	292 (43.1)	114 (16.8)	106 (15.7)
	Interrupting meals	21 (3.1)	110 (16.2)	234 (34.6)	221 (32.6)	91 (13.4)
Psychological disability	Embarrassed	61 (9.0)	62 (9.2)	241 (35.6)	230 (34.0)	83 (12.3)
	Difficulty relaxing	11 (1.6)	195 (28.8)	211 (31.2)	171 (25.3)	89 (13.1)
Social disability	Irritable with other people	27 (4.0)	100 (14.8)	333 (49.2)	142 (21.0)	75 (11.1)
	Constrains doing routine jobs	25 (3.7)	192 (28.4)	178 (26.3)	205 (30.3)	77 (11.4)
Handicap	Life is less satisfying	29 (4.3)	146 (21.6)	284 (41.9)	149 (22.0)	69 (10.2)
	Unable to function	39 (5.8)	139 (20.5)	272 (40.2)	131 (19.4)	96 (14.2)

Among the study participants, 357 (52.7%) had poor OHRQoL, which was significantly higher (positive association) among patients >50 years of age (AOR = 2.57; 95% CI = 1.65–4.08; *p* = 0.001) and those with a longer duration of diabetes (AOR = 3.31; 95% CI = 1.96–4.17; *p* = 0.017). Poor OHRQoL was significantly lower (negative association) among patients working in the private sector (AOR = 0.59; 95% CI = 0.44–0.82; *p* = 0.017), and those with a university-level or higher education (AOR = 0.71; 95% CI = 0.53–0.94; *p* = 0.006) (Table 3).

**Table 3:** Predictors of OHRQOL among diabetes patients (*n* = 677).

Characteristics	Total (*n* = 677)	OHRQOL		Binomial logistic regression	
		Poor (*n* = 357)	Good (*n* = 320)	Adjusted OR [AOR] (95% CI)	*p* value
**Age group**					
Less than 40 years	169	112	57	Ref	
40 to 50 years	299	162	137	1.67 (1.10–2.54)	0.016*
More than 50 years	209	83	126	2.57 (1.65–4.08)	0.001*
**Gender**					
Male	358	173	185	Ref	0.262
Female	319	184	135	0.82 (0.58–1.16)	
**Occupation** ^**#**^					
Government	171	80	91	Ref	
Private	388	214	174	0.59 (0.44–0.82)	0.017*
Unemployed	118	63	55	0.67 (0.38–1.18)	0.162
**Education level**					
Up to high school	257	120	137	Ref	0.006*
University (UG and PG)	420	237	183	0.71 (0.53–0.94)	
**Marital status**					
Married	603	321	282	Ref	0.713
Single	74	36	38	1.31 (0.48–1.66)	
**Monthly income (1 USD = 3.75 SAR)**					
Up to 5,000 SAR	109	70	39	Ref	
5,000 to 7,000 SAR	283	188	95	0.96 (0.55–1.68)	0.896
More than 7,000 SAR	285	99	186	0.81 (0.58–1.62)	0.071
**Presence of other chronic illness**					
No	454	234	220	Ref	
Yes	223	123	100	0.84 (0.57–1.22)	0.353
Duration of diabetes	8.32 ± 4.5			3.31 (1.96–4.17)	0.001*
**Smoking status (including shisha)**					
No	531	281	250	Ref	0.327
Yes	146	76	70	0.81 (0.53–1.24)	
**Dental health checkup by the dentist (every 6 months)**					
No	161	110	51	Ref	
Yes	516	247	269	2.85 (1.76–3.98)	0.007*

* Significant (*p* < 0.05) values.

# Government – Currently working in any of the government sector departments of KSA; private - currently working in private sectors, self-employed/business; Unemployed – Currently not working in any job.

The distribution of mental health symptoms in patients with T2DM, based on the results of the DASS-21, are presented in Table 4. Some form (mild, moderate, severe, or extremely severe) of depression, anxiety, and/or stress were observed in 59.7, 71.1, and 67.1% of all included participants, respectively.

**Table 4:** Mental health status of the participants assessed by the Depression, Anxiety and Stress Scale – 21 (DASS – 21) (*n* = 677).

Status	Depression *n* (%)	Anxiety *n* (%)	Stress *n* (%)
Normal	273 (40.3)	196 (28.9)	223 (32.9)
Mild	171 (25.3)	241 (35.6)	179 (26.4)
Moderate	130 (19.2)	171 (25.3)	144 (21.3)
Severe	63 (9.3)	27 (4.0)	62 (9.2)
Extremely severe	40 (5.9)	42 (6.2)	69 (10.2)

The DASS-21 depression subscale scores were significantly associated with gender (*p* = 0.001), occupation (*p* = 0.045), and smoking status (*p* = 0.017); the anxiety subscale scores were significantly associated with age (*p* = 0.001), gender (*p* < 0.001), education status (0.029), and presence of another chronic illness (*p* = 0.001); the stress subscale was significantly related to gender (*p* < 0.001), work status (*p* = 0.001), and smoking status (*p* = 0.001) (Table 5).

**Table 5:** Patients characteristics and its association with total and each subscale of DASS-21 (*n* = 677).

Variables	DASS 21 – Total		Depression		Anxiety		Stress	
	Median (IQR)	Value of *p*	Median (IQR)	Value of *p*	Median (IQR)	Value of *p*	Median (IQR)	Value of *p*
Overall	27 (11)		10 (3)		9 (4)		9 (5)	
Age group**								
Less than 40 years	26 (12)	0.006*	9 (5)	0.200	10 (4)	0.001*	8 (6)	0.063
40 to 50 years	28 (11)		9 (4)		9 (3)		9 (6)	
More than 50 years	28 (7)		10 (3)		8 (4)		10 (5)	
Gender***								
Male	29 (9)	0.001*	10 (3)	0.001*	10 (4)	<0.001*	10 (5)	<0.001*
Female	27 (10)		9 (3)		9 (5)		8 (5)	
**Occupation** ^**#/**^ **								
Government	27 (13)	0.038*	8 (4)	0.045*	8 (7)	0.173	9 (5)	0.001*
Private unemployed	27 (9)		10 (3)		10 (4)		8 (4)	
	33 (10)		10 (4)		9 (5)		12 (4)	
Education level***								
Up to high school	27 (11)	0.309	10 (4)	0.547	10 (5)	0.029*	9 (6)	0.217
University (UG and PG)	27 (10)		10 (3)		9 (3)		9 (6)	
Marital status***								
Married	27 (10)	0.012*	9 (3)	0.249	9 (4)	0.081	10 (3)	0.181
Single	28 (9)		10 (4)		9 (3)		9 (5)	
Monthly income in SAR (1 USD = 3.75 SAR)**								
Up to 5,000	27 (8)	0.093	8 (4)	0.104	10 (5)	0.298	10 (5)	0.031*
5,000 to 7,000	27 (10)		10 (4)		9 (4)		8 (6)	
More than 7,000	28 (10)		10 (3)		10 (4)		8 (5)	
Presence of chronic illness***								
No	28 (7)	0.347	9 (3)	0.217	10 (4)	0.001*	10 (5)	0.027*
Yes	30 (12)		10 (5)		9 (5)		10 (4)	
Smoking status (including shisha)***								
No	27 (10)	0.017*	8 (6)	0.240	9 (4)	0.179	8 (5)	0.001*
Yes	32 (12)		10 (3)		11 (4)		11 (7)	

*Significant value, **Test applied: Kruskal Wallis, ***Test applied: Mann–Whitney U test. ^#^Government – Currently working in any of the government sector departments of KSA; private - currently working in private sectors, self-employed/business; Unemployed – Currently not working in any job.

Spearman’s correlation test revealed a significant positive correlation between the OHIP-14 score and the total DASS-21 score (rho = 0.247; *p* < 0.001), as well as the depression (rho = 0.251; *p* < 0.001), anxiety (rho = 0.188; *p* = 0.001), and stress (rho = 0.152; *p* = 0.003) subscales (Table 6).

**Table 6:** Correlation between OHIP-14 scores with the total and each subscale of DASS-21 (*n* = 677).

Correlation variables	Spearman’s rho value	*p* – value*
OHIP-14 and depression	0.251	<0.001
OHIP-14 and anxiety	0.188	0.001
OHIP-14 and stress	0.152	0.003
OHIP-14 and DASS-21 total score	0.247	<0.001

* *p* – value less than 0.05 (two-tailed) is significant.

We applied binomial logistic regression analysis to find the predictive effect of OHRQOL on the mental health status of T2DM patients. After adjusting with other covariables of the present study, we found that the total OHRQOL scores assessed by the OHIP-14 had a significant positive association with depression (AOR = 2.32, 95% CI = 1.34–3.71, *p* = 0.001), anxiety (AOR = 1.81, 95% CI = 1.22–2.79, *p* = 0.003), and stress (AOR = 1.43, 95% CI = 1.14–2.19, *p* = 0.026) (Table 7).

**Table 7:** The predictive effect of OHRQOL on mental health status of diabetes patients (*n* = 677).

Characteristics	Logistic regression values	Depression*	Anxiety*	Stress*
OHIP-14 Score (total)	AOR**	2.32	1.81	1.43
	95% CI of AOR	1.34–3.71	1.22–2.79	1.14–2.19
	value of *p* (two-tailed)	0.001	0.003	0.026

* Normal versus other forms of mental health subscales according to the DASS-21 instrument.

** Adjusted variables: Age, gender, occupation, educational status, marital status, monthly income, presence of chronic illness, and smoking status.

## 4. Discussion

The COVID-19 pandemic has significantly impacted the short- and long-term mental health of numerous individuals, and T2DM patients are no exception. The WHO celebrated “World Mental Health Day” on October 10th, and the theme for 2022 was “Making Mental Health & Well-Being for All a Global Priority.” The WHO’s priority for global health needs can be achieved by assessing the burden of mental health issues, including in patients with T2DM (39). These statements reinforce the importance of the present study in the post-COVID-19 pandemic times, in which we evaluated the OHRQoL and mental health status of, and their associated factors among, patients with T2DM attending outpatient diabetes care centers in the Qassim region of the KSA.

Oral health status and OHRQoL are critical in evaluating the mental health status of patients with T2DM, as there is no proper mental health without good oral health (12, 40). In the present study, we found that nearly one-fourth (23.8%) of the participants did not obtain periodic check-ups by a dental care provider, and that approximately half (52.7%) of the participants had a poor OHRQoL. The results of a recent study by Kumari et al., performed in 2020, indicated that 42.5% of patients with T2DM had a low OHRQoL (41). Interestingly, a study that assessed the OHRQoL of Iranian patients before the COVID-19 pandemic showed a much lower proportion of patients with poor OHRQoL (42). The wide variation among these studies is due to differences in study settings, OHIP assessment tools, and participants’ diabetes status. The present study used the OHIP-14 questionnaire to assess adult patients with T2DM. Irrespective of the variations in the OHRQOL in different settings, Cervino et al. affirmed that the presence of DM significantly decreases the OHRQoL and psychological alteration among patients with DM (43).

The results of the present study indicated that in patients with T2DM, OHRQoL was significantly associated with age and educational status. Similar to the results of the present study, Sandberg et al. reported that age was a critical factor for poor OHRQoL in several domains among patients with T2DM who participated in their study (44). An epidemiological survey by Kakoei et al. showed an association between oral health, blood sugar levels, and OHRQoL among the participants of their study (45). The results of the present study indicated that the incidence of poor OHRQoL was significantly lower (protective factor) in highly educated T2DM patients (AOR = 0.61; 95% CI = 0.43–0.87; *p* = 0.006). Similar to the results of the present study, Kumari et al. also found a protective relationship between educational status and OHRQoL among patients with T2DM (AOR = 0.51; 95% CI = 0.27–0.97; *p* = 0.041) (41). Another predictor of poor OHRQOL in patients with T2DM was a lack of periodic oral check-ups by dental care providers. As shown by several authors, regular oral examinations and care by dental physicians might lead to improved oral health and, therefore, OHRQOL (24, 46, 47). In the present study, however, we observed that 23.8% of participants did not regularly obtain check-ups by dental care providers. Sadeghi et al. explored a similar association between OHRQOL and dental check-ups (42).

The results of the present study indicated that some form (mild, moderate, severe, and extremely severe) of depression, anxiety, and stress were observed in 59.7, 71.1, and 67.1% of patients with T2DM, respectively. Similarly, a recent study performed by Aljohani et al. in the KSA reported that a higher proportion (73%) of patients with T2DM had some degree of depression (30). Some possible explanations for these discrepant results are the length or concurrent events of the study period and the tools used to assess depression status, which varied between the studies. Aljohani et al. collected data during the height of the COVID-19 restrictions, *via* a patient health questionnaire (nine questions). A study performed by Mukrim et al. before the COVID-19 pandemic in the northern region of the KSA reported much lower levels of depression (37.4%) and anxiety (45.6%) among patients with T2DM (48). Similarly, Sharma et al. analyzed data collected before COVID-19 from patients with T2DM, and found that depression and anxiety were reported by 57.8 and 49.7% of their study participants, respectively (49). It is worth mentioning, again, that the results of the present study and those from other studies performed during and in the post-COVID-19 pandemic affirm the short- and long-term impacts of the COVID-19 pandemic, and the need for the continuous assessment of mental health status and related factors to ensure the WHO’s mental health activities targets are met (39).

The results of present study indicated that T2DM patients’ depression status was significantly associated with age, gender, duration of diabetes, and smoking status; anxiety was significantly associated with age and the presence of other chronic illnesses; and stress was significantly associated with income and smoking status. Additionally, the results of the present study indicated that the median scores of all DASS-21 subscales were considerably higher among unemployed participants. Similar to the present study, a recent study by Birhanu et al., performed in 2022, reported that female sex and duration of diabetes (>5 years) were significant predictors of depression among patients with T2DM (50). Another study, involving Malaysian patients with T2DM, found that comorbid anxiety disorders were significantly lower among older patients (AOR = 0.96; 95% CI = 0.93–0.98), and a recent cross-sectional study by Karpha et al. performed in 2022, showed that anxiety was significantly associated with marital status, educational status, and T2DM-related complications (51, 52). Similar to the result of the present study of patients with T2DM, a study conducted in Egypt during the COVID-19 pandemic found that distress among T2DM patients was significantly associated with lower income and educational status (53). Another critical finding explored in the present study was the significant positive correlation between the OHIP-14 scores and the total DASS scores, as well as all three subscales, which indicated that higher OHIP-14 scores, which were associated with a poor OHRQoL, may lead to higher odds of developing depression, anxiety, and stress. Furthermore, the present study’s multivariable analysis also confirmed the predictive effect of OHRQOL on the mental health status of diabetes patients. A study conducted by Sandberg et al. that assessed oral health and diabetes status on the health-related quality of life reported similar findings to the present study (44). A recent survey by Hajek et al. also found similar results. In their study, the participants with poor OHRQOL were likely to develop depression and anxiety among both genders (54). Another survey conducted during COVID-19, which evaluated the impact of the COVID-19 pandemic on oral health and psychological factors, stated that during COVID-19 pandemic indicated a positive association (21).

The present study evaluated the OHRQoL of patients with T2DM living in the central region of the KSA, and its association with mental health, using a standardized methodology and a validated tool. The authors would like to mention, however, the limitations of the present study. We utilized a quantitative cross-sectional study design among patients with T2DM from outpatient clinics; therefore, the results of the present study may not apply to all patients with DM. Second, the cross-sectional study design used in the present study might not have detected a causal relationship between the risk factors and outcome, or behaviors over the study period. Another limitation of the cross-sectional study protocol is that we could not identify a temporal association between OHRQOL and mental health status in patients with DM. Third, questionnaire-based survey-related biases, such as recall, exaggeration, and self-reported bias, cannot be excluded in our results. Finally, we conducted the present study based on patients living in the central portion of the KSA. The findings, therefore, may not reflect the OHRQOL and mental health status of all patients with DM in the KSA as a whole. Therefore, an exploratory prospective survey aiming to evaluate the qualitative aspects and temporal association between OHRQOL and the mental health status of T2DM and other types of diabetes patients should be conducted in all regions of the KSA.

## 5. Conclusion

The results of the present study indicated that more than half of the patients with T2DM had a poor OHRQOL, which was significantly associated with age and educational status. We identified that nearly one-fourth of the patients with T2DM obtain regular check-ups of their oral status with a dentist, and that a high proportion of participants had depression, anxiety, and/or stress. Furthermore, we found a significant positive correlation between the OHIP-14 scores and each of the DASS-21 subscales, indicating that a higher OHRQOL can improve the mental health status of patients with T2DM. The results of the present study suggest the need for appropriate and targeted health education programs for patients with T2DM on the importance of periodic dental examinations and oral health. Additionally, we recommended, during follow-up visits at outpatient diabetes care centers, that all T2DM patients seek counselling with trained healthcare providers to improve their mental health status.

## Data availability statement

The raw data supporting the conclusions of this article will be made available by the authors, without undue reservation.

## Ethics statement

The studies involving human participants were reviewed and approved by Regional Research Ethics Committee, Qassim Health Affairs. The patients/participants provided their written informed consent to participate in this study.

## Author contributions

AT, MA, MS, AHA, and LA made substantial contribution in conceptualization and design of the present study. AT, MA, BA, AAA, ASA, and RA involved in acquisition of the data. MA, AT, ASA, and LA involved in data entry. AT, MS, AHA, BA, AAA, ASA, and RA involved in analysis and interpretation of the data. AT involved in drafting the article, with all other authors involved in critically revising the manuscript. All authors approved the final version of the manuscript to be published and agreed to be held accountable for all aspects of the work.

## Conflict of interest

The authors declare that the research was conducted in the absence of any commercial or financial relationships that could be construed as a potential conflict of interest.

## Publisher’s note

All claims expressed in this article are solely those of the authors and do not necessarily represent those of their affiliated organizations, or those of the publisher, the editors and the reviewers. Any product that may be evaluated in this article, or claim that may be made by its manufacturer, is not guaranteed or endorsed by the publisher.
